# Endoscopic Full‐Thickness Plication for the Treatment of Gastroesophageal Reflux Disease: A Systematic Review and Meta‐Analysis of Randomized Sham Controlled Trials

**DOI:** 10.1002/jgh3.70056

**Published:** 2024-11-26

**Authors:** Muhammad Shahzil, Ammad Javaid Chaudhary, Ali Akram Qureshi, Fariha Hasan, Muhammad Saad Faisal, Abdullah Sohail, Muhammad Ali Khaqan, Taher Jamali, Muhammad Zarrar Khan, Eva Alsheik, Tobias Zuchelli

**Affiliations:** ^1^ Department of Internal Medicine Weiss Memorial Hospital LLC Chicago Illinois USA; ^2^ Department of Internal Medicine Henry Ford Hospital Detroit Michigan USA; ^3^ Department of Medicine King Edward Medical University Lahore Pakistan; ^4^ Department of Internal Medicine Cooper University Hospital Camden New Jersey USA; ^5^ Department of Internal Medicine University of Iowa Hospitals and Clinics Iowa City Iowa USA; ^6^ Department of Internal Medicine John H. Stroger, Jr. Hospital of Cook County Chicago Illinois USA; ^7^ Department of Gastroenterology Henry Ford Hospital Detroit Michigan USA

**Keywords:** endoscopic full‐thickness plication (EFTP), gastroesophageal reflux disease (GERD), proton pump inhibitor (PPI)

## Abstract

**Introduction:**

Gastroesophageal reflux disease (GERD) affects approximately 20% of adults in the United States. Proton pump inhibitors are the first‐line treatment but are associated with long‐term side effects. Endoscopic full‐thickness plication (EFTP) is a minimally invasive alternative that improves the valvular mechanism of the gastroesophageal junction. This meta‐analysis compared EFTP to a sham procedure for the treatment of refractory GERD.

**Materials and Methods:**

This meta‐analysis followed the Cochrane guidelines and PRISMA standards and was registered with PROSPERO (CRD42023485506). We searched MEDLINE, Embase, SCOPUS, and Cochrane Library through December 2023. Inclusion criteria targeted Randomized controlled trials comparing EFTP with sham procedures for GERD were included. Statistical analyses utilized RevMan with a random‐effects model, and the results were considered significant at *p* < 0.05.

**Results:**

Of the 2144 screened studies, three RCTs with 272 patients with GERD were included: 136 patients underwent EFTP and 136 underwent sham procedures. Primary outcomes showed a significant reduction in PPI usage (RR 0.51; 95% CI 0.35–0.73; *p* < 0.01) and more than 50% improvement in GERD‐HRQL scores at 3 months (RR 15.81; 95% CI 1.40–178.71; *p* = 0.03). No significant difference was found in the DeMeester scores (MD: 12.57; 95% CI −35.12 to 9.98; *p* = 0.27). Secondary outcomes showed no significant difference in time with esophageal pH < 4, but a significant reduction in total reflux episodes.

**Conclusions:**

EFTP significantly reduced PPI usage, improved GERD‐HRQL scores, and decreased total reflux episodes compared with sham procedures, highlighting its potential as a minimally invasive treatment. Further research is needed to compare EFTP with other minimally invasive techniques to determine the most effective treatment option.

## 1 Introduction

Gastroesophageal reflux disease (GERD) remains one of the most common chronic disorders in the general population, affecting approximately 20% of adults in the United States [1]. GERD can cause symptoms such as heartburn, regurgitation, chest pain, and cough, all of which can lead to a decline in the overall quality of life [2]. Additionally, this common disorder incurs a substantial economic burden, as evidenced by patients spending an estimated USD $5 billion annually on antireflux medications [1].

Lifestyle modifications and pharmacological interventions remain the cornerstones of GERD treatment. While proton pump inhibitor (PPI) therapy remains the first‐line treatment, these drugs require long‐term use for an adequate response and are associated with significant side effects such as increased risk of *Clostridioides difficile* infection and osteoporotic fractures [3, 4]. Surgical options for GERD can also be explored; however, they are associated with various types of morbidity and result in symptom recurrence in most patients [1]. Indeed, some patients who undergo surgery may require PPI therapy, thereby defeating the purpose of surgery [2].

Recently, minimally invasive procedures such as endoscopic full‐thickness plication (EFTP) have emerged as new alternatives to more invasive surgical options and long‐term PPI therapies for refractory GERD [4]. The EFTP technique involves administering a transmural suture at the gastroesophageal junction with the plicator system to reconfigure the gastric cardia and improve the valvular mechanism of the gastroesophageal junction [4]. Endoscopic procedures are minimally invasive and have been reported to lead to favorable outcomes, including a reduced need for PPIs [2]. However, although EFTP has logistical and therapeutic advantages, the efficacy of this technique has not yet been thoroughly investigated, and the effect of EFTP on specific patient outcomes has been explored in very few clinical studies. Given the lack of comparative data on this therapeutic approach, we performed a systematic review and meta‐analysis of currently available randomized controlled trials (RCT) that have evaluated the efficacy of the EFTP approach for managing refractory GERD compared to a sham procedure.

## 2 Materials and Methods

This meta‐analysis was conducted following the recommendations of the Cochrane Handbook for Systematic Reviews of Interventions and was reported according to the PRISMA statement [5]. This study was registered in the International Prospective Register of Systematic Reviews (PROSPERO) (CRD42023485506). Ethical approval was not required for this study.

### 2.1 Information Sources

We conducted a literature search for relevant studies in the online databases MEDLINE (via PubMed), Embase, SCOPUS, and the Cochrane Library from inception through December 2023.

### 2.2 Search Strategy

RCTs were included. The following MeSH terms and free‐text terms were used to search for the relevant articles: “EFTP,” “endoscopic full thickness plication,” “GERD,” “gastroesophageal reflux disease,” and “sham.” The detailed search strategy is provided in Table S1.

### 2.3 Eligibility Criteria

All RCTs that compared the use of EFTP for the treatment of GERD with a control sham procedure were included in the review. Studies chosen for analysis were assessed for the following inclusion parameters: (1) adult patients aged ≥ 18 years with GERD, (2) Intervention was EFTP, (3) control was a sham procedure, and (4) outcomes included post‐intervention GERD‐HRQL score, DeMeester score, total reflux episodes, and time at which esophageal pH was < 4. Abstracts were included if they met all the study criteria. Duplicate studies, case reports, case series of < 10 patients, conference proceedings, animal studies, single‐arm studies, guidelines, observational studies (case–control, retrospective/prospective cohort), unpublished articles, and reviews were excluded. If more than one publication had overlapping data, we included the most recent and updated study to capture the most comprehensive findings. Only RCTs published in English were included in this study. Geographical restrictions were not imposed. Studies lacking relevant information, with different endpoints, or without the intervention of interest (EFTP for GERD vs. sham procedure) were excluded.

### 2.4 Selection Process

All the literature search results were uploaded to Mendeley 1.19.8. After duplicate articles were removed, two reviewers (MS and AA) independently screened titles and abstracts. The remaining articles were subjected to full‐text screening to meet the inclusion criteria. Disagreements were resolved through discussion or by an independent third reviewer.

### 2.5 Data Collection Process

Two reviewers (MS and FH) independently extracted the data into an Excel spreadsheet and any discrepancies were checked and resolved by a third reviewer. Data extraction was performed according to the PICOS elements, including patient characteristics (age, sex, and weight), intervention details and comparator details (EFTP vs. sham procedure), outcome data (GERD‐HRQL score, DeMeester scores, reflux episodes, and time for which esophageal pH was < 4), baseline patient features (PPI use, Hill's grade of gastroesophageal flap valve, and esophagitis LA grade), and study characteristics (year, country/region, recruitment period, study design, and sample size).

### 2.6 Outcomes

Our primary outcomes were a reduction in PPI therapy usage, a number of patients with > 50% change in health‐related quality of life (GERD‐HRQL) scores over 3 months after the procedure and mean post‐procedure DeMeester scores. Our secondary outcomes included total post‐intervention reflux episodes (acid reflux and non‐acid reflux) and the percentage of time that esophageal pH was < 4.

### 2.7 Study Risk of Bias and Certainty of Evidence Assessment

Quality assessment of the included trials was performed using the revised “Risk of Bias tool for randomized controlled trials (RoB 2.0)” from the Cochrane Handbook for Systematic Reviews of Interventions. The studies were independently assessed by the reviewers in the following domains: randomization process, deviations from the intended interventions, missing outcome data, measurement of the outcome, and selection of the reported result. The judgments included low risk, some concerns, and a high risk of bias.

To determine the certainty of evidence for each outcome, we used the five grades of recommendation, assessment, development, and evaluation (GRADE) considerations. These considerations include study limitations, consistency of effect, imprecision, indirectness, and publication bias (GRADEpro GDT: GRADEpro Guideline Development Tool [software], McMaster University and Evidence Prime 2024, gradepro.org).

### 2.8 Statistical Analysis

Meta‐analyses were conducted using RevMan, Version 5.4 (The Cochrane Collaboration, Copenhagen, Denmark). The random effects model was used for all analyses owing to clinical heterogeneity among the selected studies. Dichotomous outcomes were reported as relative risk ratios (RR), whereas continuous endpoints reported in trials were calculated as weighted mean differences and standard deviations (SD) along with 95% confidence intervals (CI) using a random‐effects model. A *p* value < 0.05 was considered significant for all assessed outcomes. The inverse of the variance was used to provide individual weighting to the studies. The heterogeneity of the treatment effect was assessed using Cochrane's *I*‐squared statistic. Excess heterogeneity was explained using a sensitivity analysis. Publication bias could not be assessed because of the limited number of RCTs available for the analysis.

## 3 Results

### 3.1 Study Selection

**FIGURE 1:**
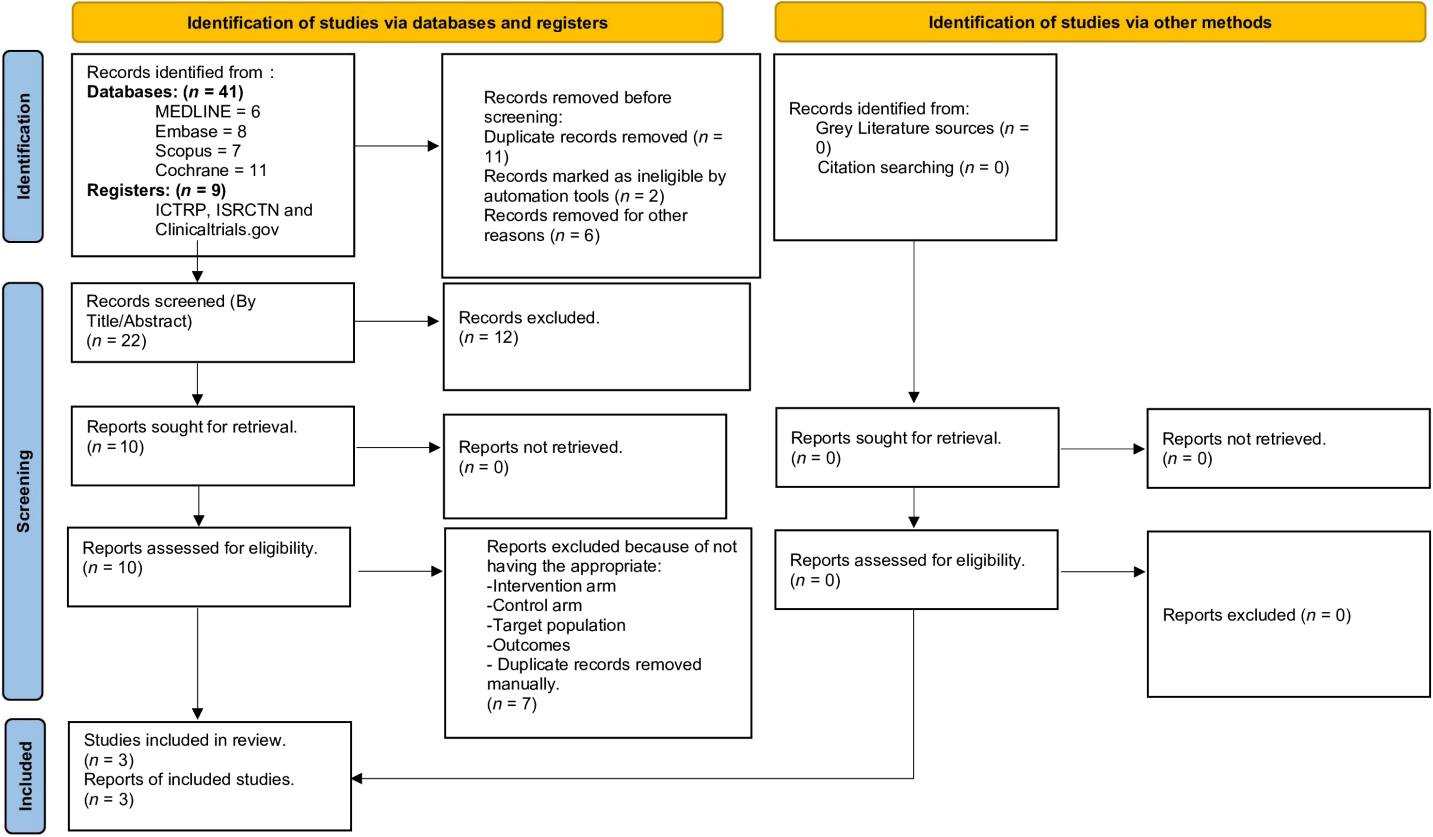
PRISMA flowchart: EFTP compared to a sham procedure.

After an extensive screening process, 50 studies were identified, and a total of 3 were included in the final analysis (Figure 1). The three studies included 289 patients with GERD. Of these, 136 (50%) were in the intervention group (EFTP) and 136 (50%) were in the control group (sham procedure). The PRISMA flowchart illustrates the study selection process (Figure 1).

### 3.2 Baseline Characteristics

Among the three RCTs, two were conducted in India [2, 6], and one was conducted in the United States and Europe [4]. All patients in all three studies were on long‐term PPI therapy. One study recruited patients with GERD who had previously undergone a peroral endoscopic myotomy (POEM) procedure [6] years for the intervention group was 43.0 ± 11.2 and 42.6 ± 11.0 years in the control group, respectively. The patients were followed up for 3–12 months. Relevant study features and baseline patient characteristics are presented in Table 1.

**TABLE 1:** Characteristics of randomized controlled trials and baseline characteristics of experimental and control groups.

	RCTs comparing EFTP vs. sham procedure for GERD					
	Maydeo et al. (2023) [6]		Kalapala et al. (2021) [2]		Rothstein et al. (2006) [4]	
PMID	36944359		33849942		16952539	
Country	India		India		USA and Europe	
Recruitment period	3 months		3 months		3 months	
Total participants	58		70		144	

	Features of experimental (EFTP) vs. control groups					
	Maydeo et al. (2023) [6]		Kalapala et al. (2021) [2]		Rothstein et al. (2006) [4]	
	EFTP	Control	EFTP	Control	EFTP	Control
Participants in group, *n*	29	29	35	35	72	72
Age, years, mean ± SD	39.9 ± 2.8	40.2 ± 2.4	35 ± 2.9	37 ± 3.8	48.1 ± 13.1	46.3 ± 13.8
Weight, lbs., mean ± SD	n.r.	n.r.	n.r.	n.r.	188.4 ± 40.6	186.2 ± 35.3
Male sex, *n* (%)	17 (56.7)	16 (53.3)	25 (71.4)	25 (71.4)	32 (45)	43 (60)
PPI use, *n* (%)	30 (100)	30 (100)	35 (100)	35 (100)	72 (100)	72 (100)
GERD HRQL, mean ± SD	10.75 ± 0.7	9.5 ± 1	38.5 ± 6.7	41.5 ± 4.8	10.9 ± 8.3	11.2 ± 9.3
DeMeester score, median (IQR)	53.9 (37.1–64.5)	52.3 (36.7–62.9)	15.1 (7.6–28.0)	12.2 (6.8–21.4)	n.d.	n.d.
% Time esophageal pH < 4 measured off PPI therapy for 7 days, median (IQR) UNITS	28.95 (2.69)	27.70 (1.83)	4.6 (1.59)	2.89 (1.09)	10 (1.68)	9.13 (1.68)
Reflux episodes, median (IQR)						
Total	148.0 (120.2–178.2)	140.5 (122.0–177.2)	90 (65–115)	92 (65–130)	n.d.	n.d.
Acid reflux	59.0 (55.7–70.5)	62.5 (50.5–68.2)	51 (33–73)	40 (27–61)	n.d.	n.d.
Nonacid reflux	89.5 (62.7–109.7)	82 (60.5–109.5)	34 (18–71)	49 (25–77)	n.d.	n.d.
Hill's grade of gastroesophageal flap valve, *n* (%)						
GRADE I	12 (40)	14 (46.7)	16 (45.7)	18 (51.4)	n.d.	n.d.
GRADE II	18 (60)	16 (53.3)	6 (17.1)	7 (20)	n.d.	n.d.
Endoscopy (esophagitis LA grade), *n* (%)						
Grade A	15 (50.0)	18 (60.0)	11 (31.4)	9 (25.7)	n.d.	n.d.
Grade B	5 (16.7)	4 (13.3)	0 (0)	1 (2.9)	n.d.	n.d.

Abbreviations: EFTP, endoscopic full‐thickness plication; GERD, gastroesophageal reflux disease; HRQL, health‐related quality of life measure; n.d., no data; PPI, proton pump inhibitor; RCT, randomized controlled trial.

### 3.3 Risk of Bias and GRADE Assessment

**FIGURE 2:**
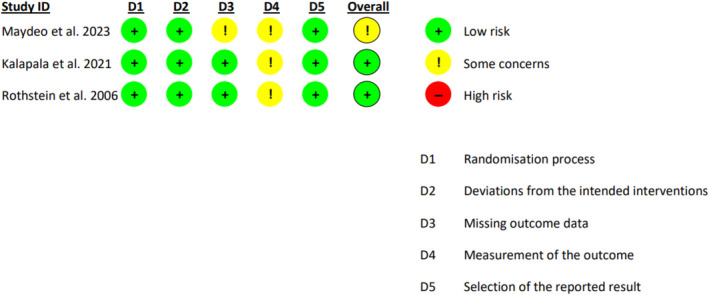
EFTP versus sham: risk of bias assessment.

Among the RCTs, two studies were low‐risk, and one study had some concerns of bias due to issues in the measurement of the outcome (Figure 2).

GRADE assessment is provided in Table S2.

### 3.4 Effects of Intervention

#### 3.4.1 Primary Outcomes

##### 3.4.1.1 Proton Pump Inhibitor (PPI) Therapy Usage

**FIGURE 3:**
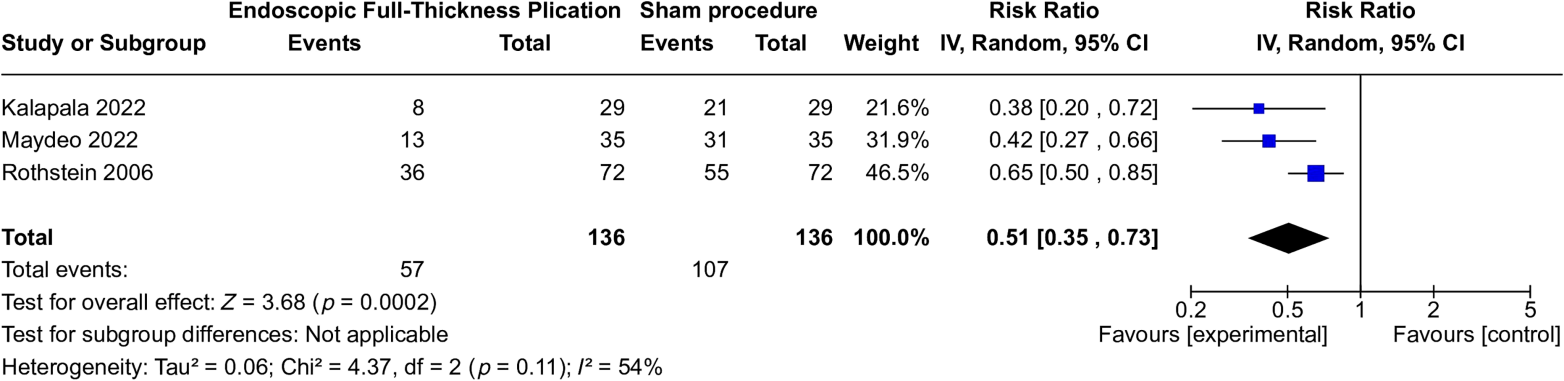
Comparison of proton pump inhibitor (PPI) therapy usage after EFTP and sham procedure.

All three studies demonstrated a reduction in PPI usage, specifically highlighting the rate at which participants in the experimental group were able to successfully discontinue PPI therapy over a follow‐up period ranging from 3 months to 1 year. The pooled analysis revealed a significantly greater reduction in PPI usage in the EFTP group compared to the sham group (RR 0.51; 95% CI 0.35–0.73; *p* = 0.0002). The studies exhibited moderate heterogeneity (*I* ^2^ = 54%). The overall quality of evidence was rated as moderate due to potential bias in the included studies (Figure 3).

##### 3.4.1.2 Number of Subjects With > 50% Improvement on GERD‐HRQL in 3 Months

**FIGURE 4:**
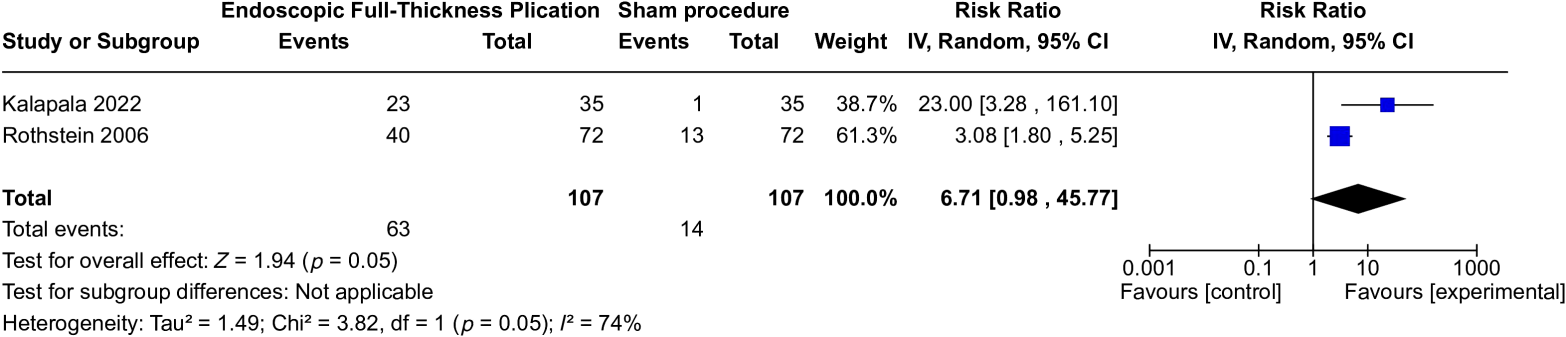
Number of subjects with > 50% GERD‐HRQL improvement post‐EFTP versus sham at 3 months.

Only two of the three studies evaluated the improvement in GERD‐HRQL scores in subjects after EFTP. The pooled analysis indicated a significantly higher number of individuals with > 50% improvement in the EFTP group than in the sham group (RR 15.81; 95% CI 1.40–178.71; *p* = 0.03). The heterogeneity observed was high (*I* ^2^ = 74%). The overall quality of evidence was rated as high due to the substantial effect (RR either > 2.0 or < 0.5, based on consistent evidence from at least two studies, with no plausible confounders) and the potential for publication bias among the included studies (Figure 4).

##### 3.4.1.3 Mean DeMeester Score

**FIGURE 5:**
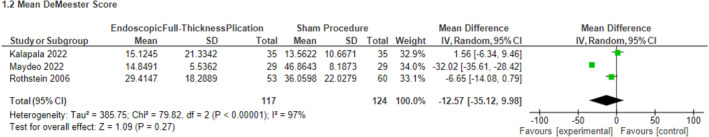
Comparison of mean DeMeester scores between EFTP and sham procedure.

All three studies reported differences in the mean DeMeester scores between the groups after the procedure. The analysis revealed no significant difference between the EFTP and sham groups (mean difference = −12.57; 95% CI −35.12 to 9.98; *p* = 0.27). The heterogeneity observed was high (*I* ^2^ = 97%). The overall quality of evidence was categorized as moderate, reflecting concerns regarding the risk of bias in the included studies (Figure 5).

#### 3.4.2 Secondary Outcomes

##### 3.4.2.1 Percentage of Time With Esophageal pH < 4

**FIGURE 6:**
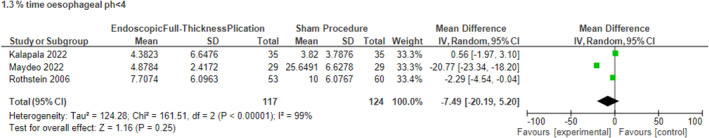
EFTP versus sham: percentage of time with esophageal pH < 4.

There were three studies that reported the percentage of time that patients had an esophageal pH < 4, with all pH testing conducted after a 7‐day cessation of PPI therapy. The analysis indicated no significant difference between the EFTP and control groups (mean difference = −7.49; 95% CI −20.19 to 5.20; *p* = 0.25). The heterogeneity observed in the studies was high (*I* ^2^ = 99%). The overall quality of evidence was deemed moderate owing to concerns about the risk of bias in the included studies (Figure 6).

##### 3.4.2.2 Total Reflux Episodes

**FIGURE 7:**
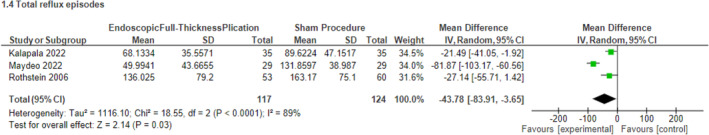
Total reflux episodes: EFTP compared to a sham procedure.

There were three studies that reported the mean number of reflux episodes after the procedure. The mean number of reflux episodes in the EFTP group was significantly lower than that in the sham treatment group (mean difference = −43.78; 95% CI −83.91 to −3.65; *p* = 0.03). The heterogeneity observed was high (*I* ^2^ = 89%). The overall quality of evidence was considered moderate, reflecting concerns regarding the risk of bias in the included studies (Figure 7).

##### 3.4.2.3 Acid Reflux Episodes

**FIGURE 8:**
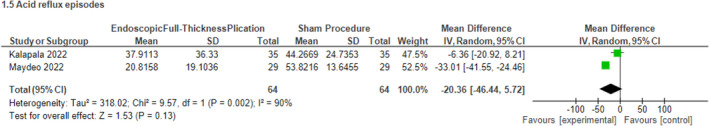
Acid reflux episodes: EFTP compared to a sham procedure.

Three studies reported the mean number of acid reflux episodes. The analysis showed no significant difference between the EFTP and control groups (mean difference = −20.36; 95% CI −46.44 to 5.72; *p* = 0.13). The heterogeneity observed was moderate (*I* ^2^ = 90%). The overall quality of evidence was rated as moderate because of concerns regarding the risk of bias and publication bias among the included studies (Figure 8).

##### 3.4.2.4 Non‐Acid Reflux Episodes

**FIGURE 9:**
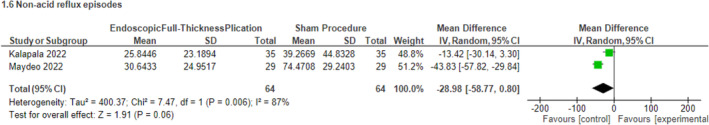
Non‐acid reflux episodes: EFTP compared to a sham procedure.

Two studies reported the mean number of non‐acid reflux episodes. The analysis indicated no significant difference between the EFTP and control groups (mean difference = −28.98; 95% CI −58.77 to 0.80; *p* = 0.06). The heterogeneity observed was moderate (*I* ^2^ = 87%). The overall quality of evidence was rated as moderate because of concerns regarding the risk of bias and publication bias among the included studies (Figure 9).

### 3.5 Sensitivity Analysis

To address the potential heterogeneity introduced by post‐POEM cases and their uncertain impact on the outcomes after EFTP, we performed a sensitivity analysis excluding the study by Maydeo et al. The exclusion had no significant effect on our overall findings, confirming the robustness of our results. Detailed results of the sensitivity analysis can be found in Appendix S2.

## 4 Discussion

To the best of our knowledge, this meta‐analysis is the first comprehensive examination of RCTs that evaluated the outcomes of patients with GERD who underwent EFTP and those who underwent a sham control procedure. Compiled findings from three RCTs affirmed the efficacy of EFTP compared to a sham procedure in reducing the need for PPIs, reducing total reflux episodes, and improving symptoms related to heartburn, as indicated by improved GERD‐HRQL scores. However, no significant differences in DeMeester scores or time with esophageal pH < 4 were observed in patients who underwent EFTP, revealing that the benefits of this procedure are likely due to a complex mechanism that requires further investigation.

Endoscopic interventions such as EFTP offer a minimally invasive option for alleviating GERD by restructuring the gastric cardia anatomy to enhance valvular function, thereby reducing reflux symptoms [7, 8]. In our study, the comparison of EFTP with a sham procedure, which serves a role analogous to a placebo, significantly reduced bias. This enhances the reliability and potential generalizability of clinical trial data. Such an approach is particularly valuable in trials involving procedures such as EFTP that have subjective endpoints, such as patient‐reported scales measuring symptoms, and provides a robust means to manage additional effects, enhancing the impartial assessment of placebo versus procedural effects [9].

Our meta‐analysis revealed a significant reduction in PPI use after adjunctive EFTP. These results align with those of previous RCTs and non‐randomized studies investigating EFTP versus alternative interventions [10, 11, 12, 13]. Given the adverse effects associated with prolonged PPI use, adopting a minimally invasive endoscopic approach is a promising new treatment option that addresses the drawbacks of PPI therapy, including daily administration, incomplete symptom relief, adverse effects, and associated costs. Harwani et al. also showed that EFTP, when combined with a single clip and argon plasma coagulation, proved to be more cost‐effective (PMID: 38813575).

Moreover, our analysis showed a significantly higher percentage of individuals experiencing > 50% improvement in GERD‐HRQL scores within 3 months of the EFTP procedure relative to control subjects. This underscores the clinical significance of endoscopic plication for managing GERD symptoms, which has a direct impact on patients' quality of life. However, one limitation was the absence of GERD‐HRQL scores in one study. Individuals with GERD often seek additional therapies to overcome PPI‐induced symptom relief, aiming for complete normalization and effective symptom control. Our findings align with those of previous studies that emphasized the key role of EFTP in improving GERD‐HRQL scores [7].

Another notable finding regarding a direct measure of symptomatic relief in patients was that patients had significantly fewer reflux episodes after EFTP than after the sham procedure, which is consistent with previous studies [14, 15]. However, our analysis revealed no significant difference between the groups in other components of the reflux symptom index, indicating that the clinical response to EFTP may be attributed to a decrease in reflux volume rather than a normalization of acid exposure time. This also aligns with previous research, which showed that decreased esophageal acid exposure after EFTP is uncommon, although it sometimes reaches normalization [16, 17]. Notably, endoscopic plication has demonstrated no significant effect on manometric features, consistent with the theoretical framework suggesting that both the lower esophageal sphincter and diaphragm contribute to maintaining gastroesophageal sphincter competence. Notably, this intervention does not induce structural alterations in the esophageal hiatus [17, 18, 19, 20].

Our analysis included EFTP procedures involving the GERDx system and the NDO Plicator, despite the unavailability of the latter device in the commercial market. This decision was justified by the shared mechanism and similar procedural processes of both devices. Both procedures are conducted under general anesthesia and employ pre‐tied transmural pledgeted sutures to create a tissue valve at the lower esophageal sphincter, reinforcing its competence [12, 13, 14, 17, 21, 22]. Regarding the various GERD therapy options, one network meta‐analysis underscored the superior effectiveness of both endoscopic and surgical interventions compared with standalone PPI therapy in enhancing GERD‐HRQL scores, which is especially relevant given the significant decline in the quality of life experienced by patients with GERD despite adherence to acid‐suppressive medications. Notably, the assessment of objective metrics, including abnormal acid exposure and lower esophageal sphincter resting pressure, showed that only surgical fundoplication was superior to PPI therapy, and patients who had radiofrequency energy delivery, endoscopic plication, and lower esophageal sphincter reinforcement showed no significant differences in these parameters [15].

Another recent network meta‐analysis found that Stretta and TIF were significantly better than PPIs at improving health‐related quality of life and reducing heartburn. While Stretta was less effective than TIF at increasing lower esophageal sphincter pressure, it was more effective at reducing esophageal acid exposure. However, both procedures were comparable in reducing PPI use and the incidence of esophagitis (PMID: 33650003). Likewise, the emerging therapy anti‐reflux mucosectomy (ARMS) has shown promise, with 40%–50% of patients discontinuing PPI use and significant improvements in symptom scores and acid exposure metrics (PMID: 32415898). Emphasizing that normalizing acid exposure is not a primary GERD management endpoint, the study authors advocate prioritizing symptomatic relief and PPI discontinuation as the most relevant outcomes for patients with GERD. The authors also distinctly positioned all endoscopic and surgical approaches (excluding radiofrequency energy delivery) as effective in reducing the need for PPI medication, particularly highlighting the superiority of surgical fundoplication. These observations underscore the need for a nuanced approach to GERD therapy, specifically endorsing endoscopic or surgical treatments for patients with PPI‐dependent or refractory GERD, which is supported by the predominant theme of most studies that have focused on this patient subset [15].

Despite the overall positive outcomes, our study has some limitations. Variability in sample sizes across studies may have impacted the individual result power and pooled analyses. Additionally, the reported outcomes differed among the studies, and the inclusion of a relatively small number of studies poses a limitation. The heterogeneity in patient populations, including those who underwent previous POEM procedures for achalasia, introduces variability in baseline characteristics. Although the sensitivity analysis did not show significant changes, the physiological differences among these populations limit our ability to fully understand the implications of the results for the general population. This diversity may impact the generalizability of the findings to different patient populations and clinical settings. The variable durations of follow‐up periods in the different trials warrant consideration, although our analysis focused on the 3‐month outcomes. Previous studies tracking the long‐term outcomes of surgical GERD treatments have suggested a notable relapse rate after 5 years [23]. Concurrently, research has demonstrated a significant decrease in average GERD‐HRQL scores in the first year after transoral incisionless fundoplication for GERD [13, 18]. However, Pleskow et al. reported that EFTP can lead to a reduction in GERD symptoms and medication use for at least 5 years after the procedure, with no observed long‐term procedure‐related adverse events [24]. Investigating long‐term outcomes following EFTP is crucial, and more RCTs with extended follow‐up are needed. While our findings support the use of EFTP for managing GERD symptoms compared to sham procedures, further research is essential. Comparative studies with other minimally invasive techniques and multicentric trials are vital to validate EFTP's efficacy and determine its relevance across diverse patient populations.

## 5 Conclusions

In conclusion, our meta‐analysis highlights the efficacy of EFTP in managing GERD symptoms compared with sham procedures. EFTP has great promise as a safe and effective approach for treating GERD, especially in reducing PPI usage, reducing reflux episodes, and improving GERD‐HRQL scores, which indicates a direct positive impact on quality of life. Our findings contribute to the evidence base, but further research comparing EFTP to other minimally invasive techniques is crucial for obtaining comprehensive evidence. Such research will continue to inform clinical decisions and help clinicians decide the most suitable treatment options tailored to individual patients.

## Conflicts of Interest

The authors declare no conflicts of interest.

## Supporting information


Appendix S1.

Tables S1–S2.

## Data Availability

The data analyzed in this study is publicly available.
