# Supplementary material for: Endoscopic Full‐Thickness Plication for the Treatment of Gastroesophageal Reflux Disease: A Systematic Review and Meta‐Analysis of Randomized Sham Controlled Trials
Source: JGH Open. 2024 Nov 26;8(11):e70056. doi: 10.1002/jgh3.70056 (PMC11599161; doi:10.1002/jgh3.70056)
Supplement: Supplementary file 1 — Appendix S1. [file JGH3-8-e70056-s002.docx]

# Sensitivity Analysis:

- 1. ***Proton Pump Inhibitor (PPI) Therapy Usage:

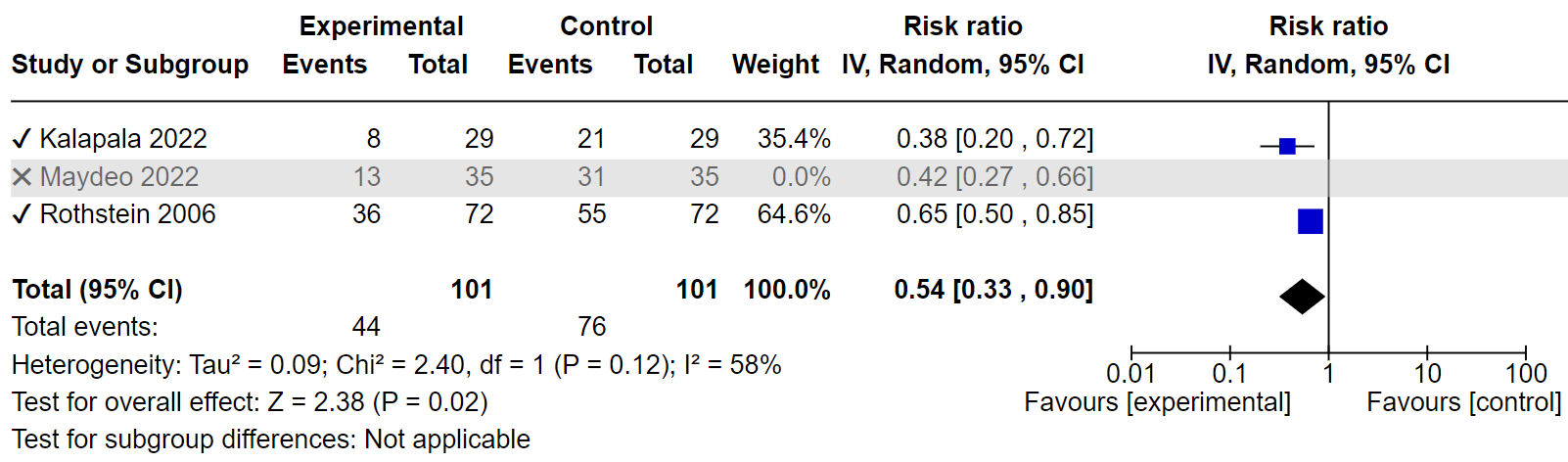
***

***1.3 Mean DeMeester Score:***

***
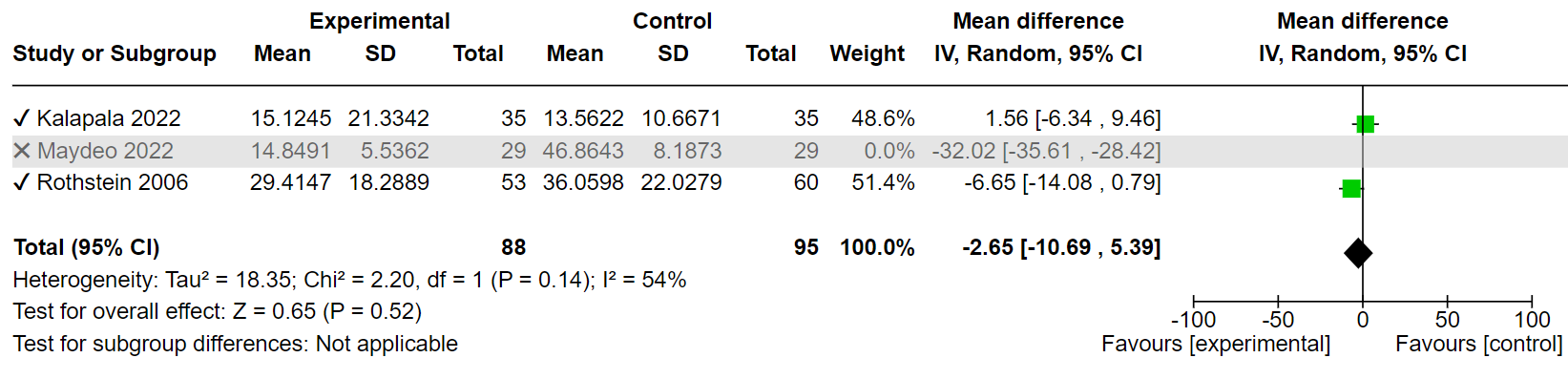
***

***2.1 Percentage of Time with Esophageal pH <4:***

***
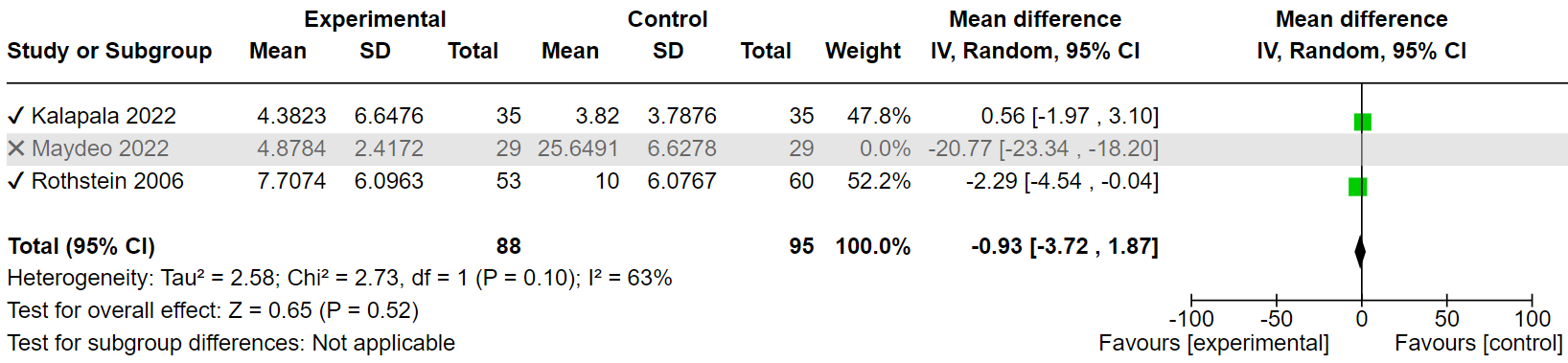
***

***2.2 Total Reflux Episodes:***

***
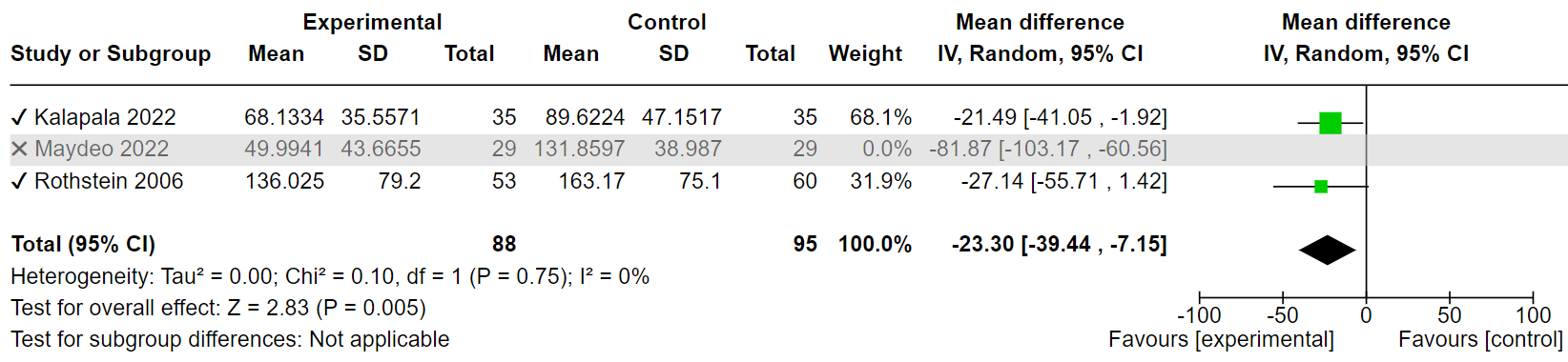
***

***2.3 Acid Reflux Episodes:***

***
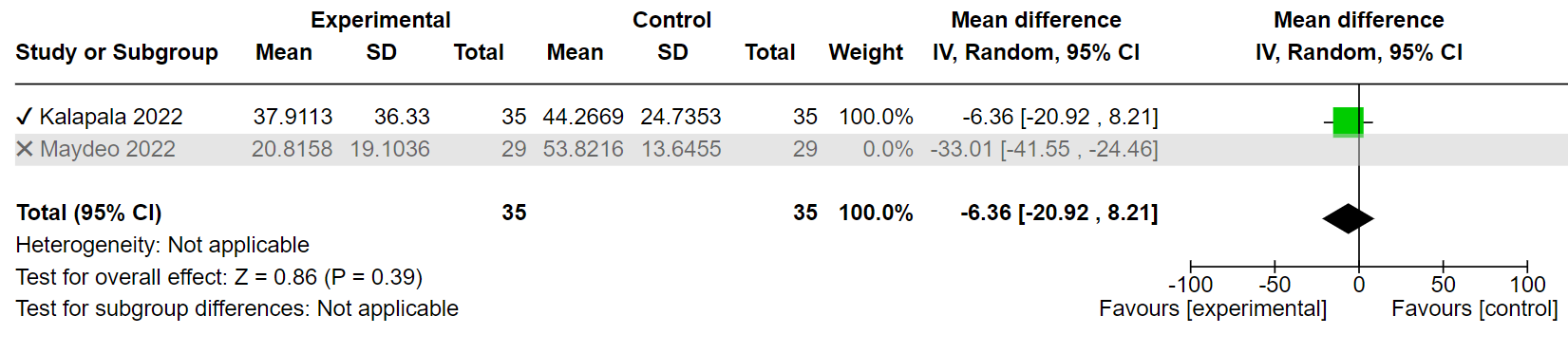
***

***2.4 Non-Acid Reflux Episodes:***

***
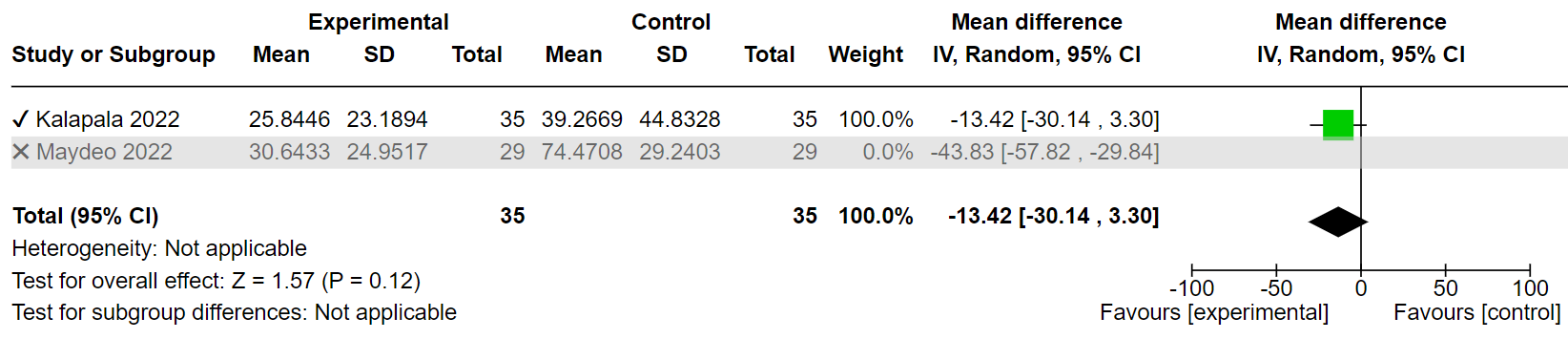
***
