# Supplementary material for: Endoscopic Full‐Thickness Plication for the Treatment of Gastroesophageal Reflux Disease: A Systematic Review and Meta‐Analysis of Randomized Sham Controlled Trials
Source: JGH Open. 2024 Nov 26;8(11):e70056. doi: 10.1002/jgh3.70056 (PMC11599161; doi:10.1002/jgh3.70056)
Supplement: Supplementary file 2 — Tables S1–S2. [file JGH3-8-e70056-s001.docx]

Table S1. Full search terms

| Number | Search Terms |
| --- | --- |
| #1 | Endoscopic Full Thickness Plication [All Fields] |
| #2 | EFTP [All Fields] |
| #3 | #1 OR #2 |
| #4 | Gastroesophageal Reflux* [All Fields] |
| #5 | GERD [All Fields] |
| #6 | #4 OR #5 |
| #7 | Sham* [All Fields] |
| #8 | #3 AND #6 AND #7 |

Table S2

Certainty of Evidence for Endoscopic Full-Thickness Plication vs. Sham Procedure in GERD Outcomes.

| **Certainty assessment** | | | | | | | **№ of patients** | | **Effect** | | **Certainty** | **Importance** |
| --- | --- | --- | --- | --- | --- | --- | --- | --- | --- | --- | --- | --- |
| **№ of studies** | **Study design** | **Risk of bias** | **Inconsistency** | **Indirectness** | **Imprecision** | **Other considerations** | **ENDOSCOPIC FULL-THICKNESS PLICATION** | **Sham Procedure** | **Relative (95% CI)** | **Absolute (95% CI)** |  |  |
| **Mean DeMeester Score.** | | | | | | | | | | | | |
| 3 | randomised trials | serious | not serious | not serious | not serious | none | 117 | 124 | - | MD **12.57 Percentage lower** (35.12 lower to 9.98 higher) | ⨁⨁⨁◯ Moderate |  |
| **Number of people with >50% improvement on GERD-HRQL in 3 month** | | | | | | | | | | | | |
| 2 | randomised trials | not serious | not serious | not serious | not serious | publication bias strongly suspected strong association | 63/107 (58.9%) | 14/107 (13.1%) | **RR 6.71** (0.98 to 45.77) | **747 more per 1,000** (from 3 fewer to 1,000 more) | ⨁⨁⨁⨁ High |  |
| **% time eoesophageal ph<4** | | | | | | | | | | | | |
| 3 | randomised trials | serious | not serious | not serious | not serious | none | 117 | 124 | - | MD **5.2 Percentage higher** (20.19 lower to 5.2 higher) | ⨁⨁⨁◯ Moderate |  |
| **Total reflux episodes** | | | | | | | | | | | | |
| 3 | randomised trials | serious | not serious | not serious | not serious | none | 117 | 124 | - | MD **27.14 lower** (55.71 lower to 1.42 higher) | ⨁⨁⨁◯ Moderate |  |
| **Acid reflux episodes** | | | | | | | | | | | | |
| 2 | randomised trials | not serious | not serious | not serious | not serious | publication bias strongly suspected | 64 | 64 | - | MD **20.36 lower** (46.44 lower to 5.72 higher) | ⨁⨁⨁◯ Moderate |  |
| **Non-acid reflux episodes** | | | | | | | | | | | | |
| 2 | randomised trials | not serious | not serious | not serious | not serious | publication bias strongly suspected | 64 | 64 | - | MD **28.98 lower** (58.77 lower to 0.8 higher) | ⨁⨁⨁◯ Moderate |  |
| **Usage of PPI Follow-up after the study** | | | | | | | | | | | | |
| 3 | randomised trials | serious | not serious | not serious | not serious | none | 57/136 (41.9%) | 107/136 (78.7%) | **RR 0.51** (0.35 to 0.73) | **386 fewer per 1,000** (from 511 fewer to 212 fewer) | ⨁⨁⨁◯ Moderate |  |
